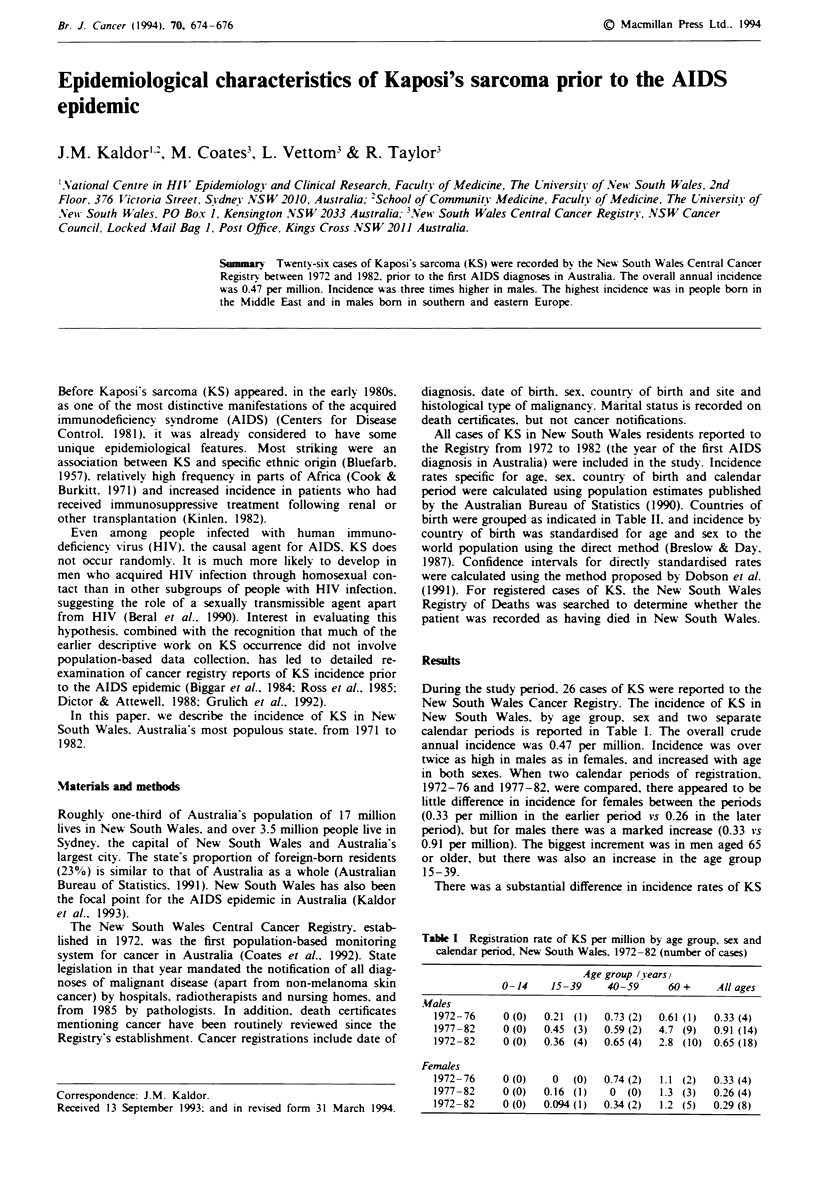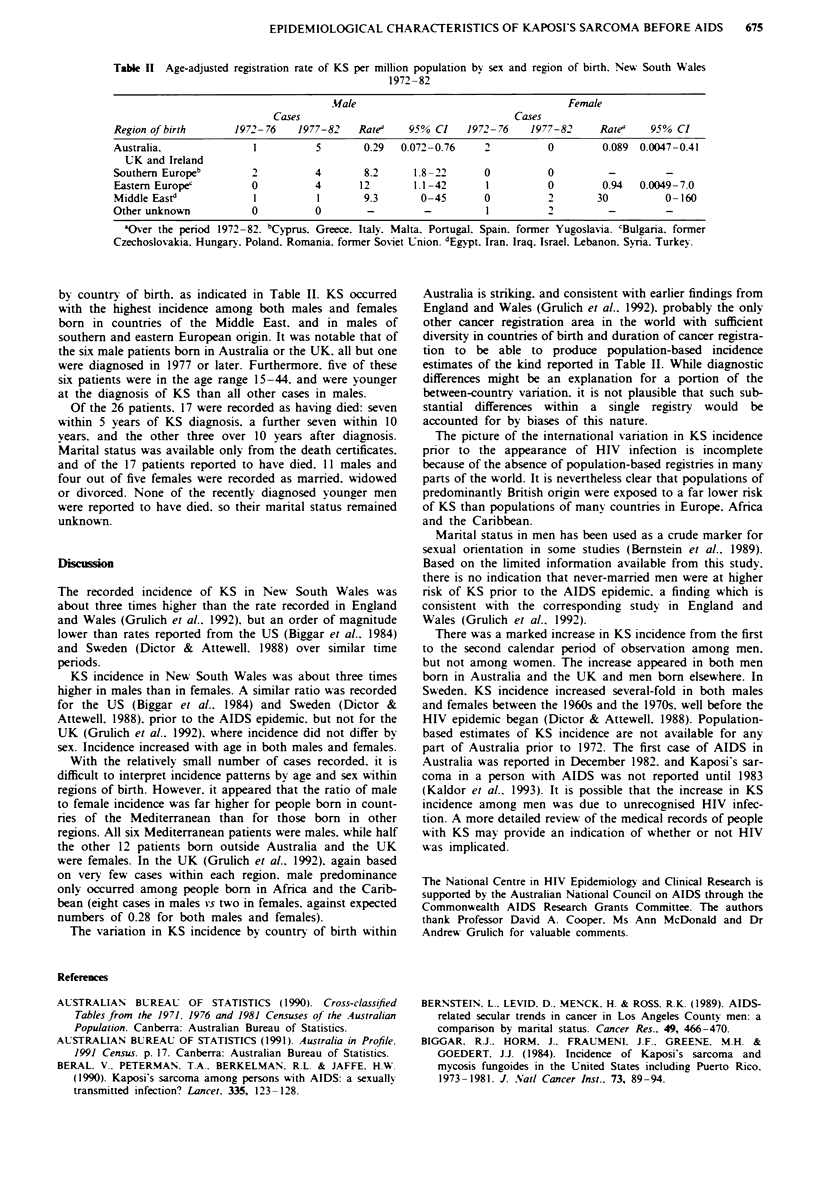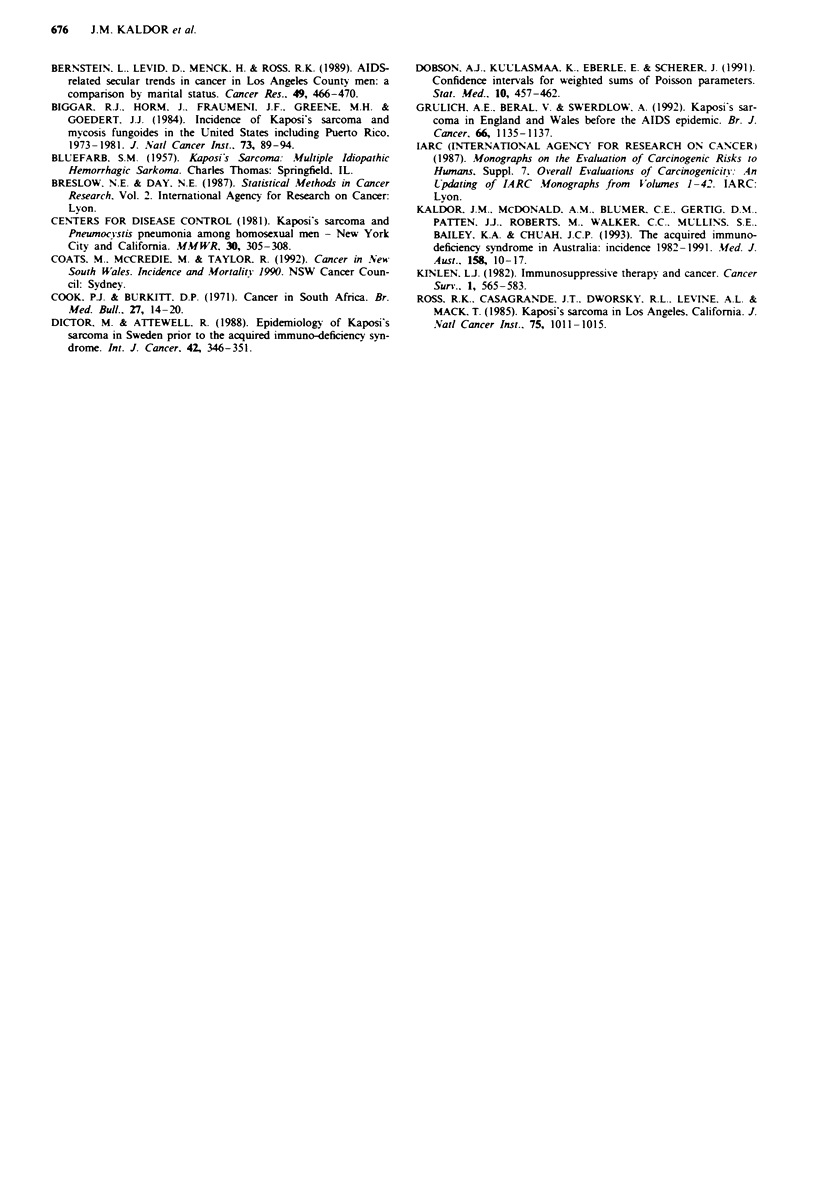# Epidemiological characteristics of Kaposi's sarcoma prior to the AIDS epidemic.

**DOI:** 10.1038/bjc.1994.370

**Published:** 1994-10

**Authors:** J. M. Kaldor, M. Coates, L. Vettom, R. Taylor

**Affiliations:** National Centre in HIV Epidemiology and Clinical Research, Faculty of Medicine, University of New South Wales, Sydney, Australia.

## Abstract

Twenty-six cases of Kaposi's sarcoma (KS) were recorded by the New South Wales Central Cancer Registry between 1972 and 1982, prior to the first AIDS diagnoses in Australia. The overall annual incidence was 0.47 per million. Incidence was three times higher in males. The highest incidence was in people born in the Middle East and in males born in southern and eastern Europe.


Br. J. Cancer (1994), 70, 674-676

© Macmillan Press Ltd., 1994

### Epidemiological characteristics of Kaposi's sarcoma prior to the AIDS epidemic

J.M. Kaldor[1,2], M. Coates[3], L. Vettom[3] & R. Taylor[3]

[1]*National Centre in HIV Epidemiology and Clinical Research, Faculty of Medicine, The University of New South Wales, 2nd Floor, 376 Victoria Street, Sydney NSW 2010, Australia;* [2]*School of Community Medicine, Faculty of Medicine, The University of New South Wales, PO Box 1, Kensington NSW 2033 Australia;* [3]*New South Wales Central Cancer Registry, NSW Cancer Council, Locked Mail Bag 1, Post Office, Kings Cross NSW 2011 Australia.*

**Summary** Twenty-six cases of Kaposi's sarcoma (KS) were recorded by the New South Wales Central Cancer Registry between 1972 and 1982, prior to the first AIDS diagnoses in Australia. The overall annual incidence was 0.47 per million. Incidence was three times higher in males. The highest incidence was in people born in the Middle East and in males born in southern and eastern Europe.

Before Kaposi's sarcoma (KS) appeared, in the early 1980s, as one of the most distinctive manifestations of the acquired immunodeficiency syndrome (AIDS) (Centers for Disease Control, 1981), it was already considered to have some unique epidemiological features. Most striking were an association between KS and specific ethnic origin (Bluefarb, 1957), relatively high frequency in parts of Africa (Cook & Burkitt, 1971) and increased incidence in patients who had received immunosuppressive treatment following renal or other transplantation (Kinlen, 1982).

Even among people infected with human immunodeficiency virus (HIV), the causal agent for AIDS, KS does not occur randomly. It is much more likely to develop in men who acquired HIV infection through homosexual contact than in other subgroups of people with HIV infection, suggesting the role of a sexually transmissible agent apart from HIV (Beral *et al.*, 1990). Interest in evaluating this hypothesis, combined with the recognition that much of the earlier descriptive work on KS occurrence did not involve population-based data collection, has led to detailed reexamination of cancer registry reports of KS incidence prior to the AIDS epidemic (Biggar *et al.*, 1984; Ross *et al.*, 1985; Dictor & Attewell, 1988; Grulich *et al.*, 1992).

In this paper, we describe the incidence of KS in New South Wales, Australia's most populous state, from 1971 to 1982.

### Materials and methods

Roughly one-third of Australia's population of 17 million lives in New South Wales, and over 3.5 million people live in Sydney, the capital of New South Wales and Australia's largest city. The state's proportion of foreign-born residents (23%) is similar to that of Australia as a whole (Australian Bureau of Statistics, 1991). New South Wales has also been the focal point for the AIDS epidemic in Australia (Kaldor *et al.*, 1993).

The New South Wales Central Cancer Registry, established in 1972, was the first population-based monitoring system for cancer in Australia (Coates *et al.*, 1992). State legislation in that year mandated the notification of all diagnoses of malignant disease (apart from non-melanoma skin cancer) by hospitals, radiotherapists and nursing homes, and from 1985 by pathologists. In addition, death certificates mentioning cancer have been routinely reviewed since the Registry's establishment. Cancer registrations include date of diagnosis, date of birth, sex, country of birth and site and histological type of malignancy. Marital status is recorded on death certificates, but not cancer notifications.

All cases of KS in New South Wales residents reported to the Registry from 1972 to 1982 (the year of the first AIDS diagnosis in Australia) were included in the study. Incidence rates specific for age, sex, country of birth and calendar period were calculated using population estimates published by the Australian Bureau of Statistics (1990). Countries of birth were grouped as indicated in Table II, and incidence by country of birth was standardised for age and sex to the world population using the direct method (Breslow & Day, 1987). Confidence intervals for directly standardised rates were calculated using the method proposed by Dobson *et al.* (1991). For registered cases of KS, the New South Wales Registry of Deaths was searched to determine whether the patient was recorded as having died in New South Wales.

### Results

During the study period, 26 cases of KS were reported to the New South Wales Cancer Registry. The incidence of KS in New South Wales, by age group, sex and two separate calendar periods is reported in Table I. The overall crude annual incidence was 0.47 per million. Incidence was over twice as high in males as in females, and increased with age in both sexes. When two calendar periods of registration, 1972-76 and 1977-82, were compared, there appeared to be little difference in incidence for females between the periods (0.33 per million in the earlier period *vs* 0.26 in the later period), but for males there was a marked increase (0.33 *vs* 0.91 per million). The biggest increment was in men aged 65 or older, but there was also an increase in the age group 15-39.

There was a substantial difference in incidence rates of KS

**Table I** Registration rate of KS per million by age group, sex and calendar period, New South Wales, 1972-82 (number of cases)

| | *Age group (years)* | | | | |
|---|---|---|---|---|---|
| | *0-14* | *15-39* | *40-59* | *60+* | *All ages* |
| *Males* | | | | | |
| 1972-76 | 0 (0) | 0.21 (1) | 0.73 (2) | 0.61 (1) | 0.33 (4) |
| 1977-82 | 0 (0) | 0.45 (3) | 0.59 (2) | 4.7 (9) | 0.91 (14) |
| 1972-82 | 0 (0) | 0.36 (4) | 0.65 (4) | 2.8 (10) | 0.65 (18) |
| *Females* | | | | | |
| 1972-76 | 0 (0) | 0 (0) | 0.74 (2) | 1.1 (2) | 0.33 (4) |
| 1977-82 | 0 (0) | 0.16 (1) | 0 (0) | 1.3 (3) | 0.26 (4) |
| 1972-82 | 0 (0) | 0.094 (1) | 0.34 (2) | 1.2 (5) | 0.29 (8) |

Correspondence: J.M. Kaldor.
Received 13 September 1993; and in revised form 31 March 1994.

**Table II** Age-adjusted registration rate of KS per million population by sex and region of birth, New South Wales 1972-82

| | *Male* | | | | *Female* | | | |
|---|---|---|---|---|---|---|---|---|
| | *Cases* | | | | *Cases* | | | |
| *Region of birth* | *1972-76* | *1977-82* | *Rate*[a] | *95% CI* | *1972-76* | *1977-82* | *Rate*[a] | *95% CI* |
| Australia, UK and Ireland | 1 | 5 | 0.29 | 0.072-0.76 | 2 | 0 | 0.089 | 0.0047-0.41 |
| Southern Europe[b] | 2 | 4 | 8.2 | 1.8-22 | 0 | 0 | – | – |
| Eastern Europe[c] | 0 | 4 | 12 | 1.1-42 | 1 | 0 | 0.94 | 0.0049-7.0 |
| Middle East[d] | 1 | 1 | 9.3 | 0-45 | 0 | 2 | 30 | 0-160 |
| Other unknown | 0 | 0 | – | – | 1 | 2 | – | – |

[a]Over the period 1972-82. [b]Cyprus, Greece, Italy, Malta, Portugal, Spain, former Yugoslavia. [c]Bulgaria, former Czechoslovakia, Hungary, Poland, Romania, former Soviet Union. [d]Egypt, Iran, Iraq, Israel, Lebanon, Syria, Turkey.

by country of birth, as indicated in Table II. KS occurred with the highest incidence among both males and females born in countries of the Middle East, and in males of southern and eastern European origin. It was notable that of the six male patients born in Australia or the UK, all but one were diagnosed in 1977 or later. Furthermore, five of these six patients were in the age range 15-44, and were younger at the diagnosis of KS than all other cases in males.

Of the 26 patients, 17 were recorded as having died: seven within 5 years of KS diagnosis, a further seven within 10 years, and the other three over 10 years after diagnosis. Marital status was available only from the death certificates, and of the 17 patients reported to have died, 11 males and four out of five females were recorded as married, widowed or divorced. None of the recently diagnosed younger men were reported to have died, so their marital status remained unknown.

### Discussion

The recorded incidence of KS in New South Wales was about three times higher than the rate recorded in England and Wales (Grulich *et al.*, 1992), but an order of magnitude lower than rates reported from the US (Biggar *et al.*, 1984) and Sweden (Dictor & Attewell, 1988) over similar time periods.

KS incidence in New South Wales was about three times higher in males than in females. A similar ratio was recorded for the US (Biggar *et al.*, 1984) and Sweden (Dictor & Attewell, 1988), prior to the AIDS epidemic, but not for the UK (Grulich *et al.*, 1992), where incidence did not differ by sex. Incidence increased with age in both males and females.

With the relatively small number of cases recorded, it is difficult to interpret incidence patterns by age and sex within regions of birth. However, it appeared that the ratio of male to female incidence was far higher for people born in countries of the Mediterranean than for those born in other regions. All six Mediterranean patients were males, while half the other 12 patients born outside Australia and the UK were females. In the UK (Grulich *et al.*, 1992), again based on very few cases within each region, male predominance only occurred among people born in Africa and the Caribbean (eight cases in males *vs* two in females, against expected numbers of 0.28 for both males and females).

The variation in KS incidence by country of birth within Australia is striking, and consistent with earlier findings from England and Wales (Grulich *et al.*, 1992), probably the only other cancer registration area in the world with sufficient diversity in countries of birth and duration of cancer registration to be able to produce population-based incidence estimates of the kind reported in Table II. While diagnostic differences might be an explanation for a portion of the between-country variation, it is not plausible that such substantial differences within a single registry would be accounted for by biases of this nature.

The picture of the international variation in KS incidence prior to the appearance of HIV infection is incomplete because of the absence of population-based registries in many parts of the world. It is nevertheless clear that populations of predominantly British origin were exposed to a far lower risk of KS than populations of many countries in Europe, Africa and the Caribbean.

Marital status in men has been used as a crude marker for sexual orientation in some studies (Bernstein *et al.*, 1989). Based on the limited information available from this study, there is no indication that never-married men were at higher risk of KS prior to the AIDS epidemic, a finding which is consistent with the corresponding study in England and Wales (Grulich *et al.*, 1992).

There was a marked increase in KS incidence from the first to the second calendar period of observation among men, but not among women. The increase appeared in both men born in Australia and the UK and men born elsewhere. In Sweden, KS incidence increased several-fold in both males and females between the 1960s and the 1970s, well before the HIV epidemic began (Dictor & Attewell, 1988). Population-based estimates of KS incidence are not available for any part of Australia prior to 1972. The first case of AIDS in Australia was reported in December 1982, and Kaposi's sarcoma in a person with AIDS was not reported until 1983 (Kaldor *et al.*, 1993). It is possible that the increase in KS incidence among men was due to unrecognised HIV infection. A more detailed review of the medical records of people with KS may provide an indication of whether or not HIV was implicated.

The National Centre in HIV Epidemiology and Clinical Research is supported by the Australian National Council on AIDS through the Commonwealth AIDS Research Grants Committee. The authors thank Professor David A. Cooper, Ms Ann McDonald and Dr Andrew Grulich for valuable comments.

### References

AUSTRALIAN BUREAU OF STATISTICS (1990). *Cross-classified Tables from the 1971, 1976 and 1981 Censuses of the Australian Population.* Canberra: Australian Bureau of Statistics.

AUSTRALIAN BUREAU OF STATISTICS (1991). *Australia in Profile, 1991 Census.* p. 17. Canberra: Australian Bureau of Statistics.

BERAL, V., PETERMAN, T.A., BERKELMAN, R.L. & JAFFE, H.W. (1990). Kaposi's sarcoma among persons with AIDS: a sexually transmitted infection? *Lancet*, **335**, 123-128.

BERNSTEIN, L., LEVID, D., MENCK, H. & ROSS, R.K. (1989). AIDS-related secular trends in cancer in Los Angeles County men: a comparison by marital status. *Cancer Res.*, **49**, 466-470.

BIGGAR, R.J., HORM, J., FRAUMENI, J.F., GREENE, M.H. & GOEDERT, J.J. (1984). Incidence of Kaposi's sarcoma and mycosis fungoides in the United States including Puerto Rico, 1973-1981. *J. Natl Cancer Inst.*, **73**, 89-94.

BERNSTEIN, L., LEVID, D., MENCK, H. & ROSS, R.K. (1989). AIDS-related secular trends in cancer in Los Angeles County men: a comparison by marital status. *Cancer Res.*, **49**, 466–470.

BIGGAR, R.J., HORM, J., FRAUMENI, J.F., GREENE, M.H. & GOEDERT, J.J. (1984). Incidence of Kaposi's sarcoma and mycosis fungoides in the United States including Puerto Rico, 1973–1981. *J. Natl Cancer Inst.*, **73**, 89–94.

BLUEFARB, S.M. (1957). *Kaposi's Sarcoma: Multiple Idiopathic Hemorrhagic Sarkoma*. Charles Thomas: Springfield, IL.

BRESLOW, N.E. & DAY, N.E. (1987). *Statistical Methods in Cancer Research*, Vol. 2. International Agency for Research on Cancer: Lyon.

CENTERS FOR DISEASE CONTROL (1981). Kaposi's sarcoma and *Pneumocystis* pneumonia among homosexual men – New York City and California. *MMWR*, **30**, 305–308.

COATS, M., MCCREDIE, M. & TAYLOR, R. (1992). *Cancer in New South Wales. Incidence and Mortality 1990*. NSW Cancer Council: Sydney.

COOK, P.J. & BURKITT, D.P. (1971). Cancer in South Africa. *Br. Med. Bull.*, **27**, 14–20.

DICTOR, M. & ATTEWELL, R. (1988). Epidemiology of Kaposi's sarcoma in Sweden prior to the acquired immuno-deficiency syndrome. *Int. J. Cancer*, **42**, 346–351.

DOBSON, A.J., KUULASMAA, K., EBERLE, E. & SCHERER, J. (1991). Confidence intervals for weighted sums of Poisson parameters. *Stat. Med.*, **10**, 457–462.

GRULICH, A.E., BERAL, V. & SWERDLOW, A. (1992). Kaposi's sarcoma in England and Wales before the AIDS epidemic. *Br. J. Cancer*, **66**, 1135–1137.

IARC (INTERNATIONAL AGENCY FOR RESEARCH ON CANCER) (1987). *Monographs on the Evaluation of Carcinogenic Risks to Humans*, Suppl. 7, *Overall Evaluations of Carcinogenicity: An Updating of IARC Monographs from Volumes 1–42*. IARC: Lyon.

KALDOR, J.M., MCDONALD, A.M., BLUMER, C.E., GERTIG, D.M., PATTEN, J.J., ROBERTS, M., WALKER, C.C., MULLINS, S.E., BAILEY, K.A. & CHUAH, J.C.P. (1993). The acquired immunodeficiency syndrome in Australia: incidence 1982–1991. *Med. J. Aust.*, **158**, 10–17.

KINLEN, L.J. (1982). Immunosuppressive therapy and cancer. *Cancer Surv.*, **1**, 565–583.

ROSS, R.K., CASAGRANDE, J.T., DWORSKY, R.L., LEVINE, A.L. & MACK, T. (1985). Kaposi's sarcoma in Los Angeles, California. *J. Natl Cancer Inst.*, **75**, 1011–1015.